# Enhancing protein backbone angle prediction by using simpler models of deep neural networks

**DOI:** 10.1038/s41598-020-76317-6

**Published:** 2020-11-10

**Authors:** Fereshteh Mataeimoghadam, M. A. Hakim Newton, Abdollah Dehzangi, Abdul Karim, B. Jayaram, Shoba Ranganathan, Abdul Sattar

**Affiliations:** 1 School of Information and Communication Technology, Griffith University, Nathan, QLD Australia; 2 Institute of Integrated and Intelligent Systems, Griffith University, Nathan, QLD Australia; 3 Department of Computer Science, Rutgers University, Camden, NJ USA; 4 Center for Computational and Integrative Biology, Rutgers University, Camden, NJ USA; 5 Department of Chemistry and School of Biological Sciences, IIT Delhi, Delhi, India; 6 Department of Chemistry and Biomolecular Sciences, Macquarie University, Macquarie Park, NSW Australia

**Keywords:** Machine learning, Protein structure predictions

## Abstract

Protein structure prediction is a grand challenge. Prediction of protein structures via the representations using backbone dihedral angles has recently achieved significant progress along with the on-going surge of deep neural network (DNN) research in general. However, we observe that in the protein backbone angle prediction research, there is an overall trend to employ more and more complex neural networks and then to throw more and more features to the neural networks. While more features might add more predictive power to the neural network, we argue that redundant features could rather clutter the scenario and more complex neural networks then just could counterbalance the noise. From artificial intelligence and machine learning perspectives, problem representations and solution approaches do mutually interact and thus affect performance. We also argue that comparatively simpler predictors can more easily be reconstructed than the more complex ones. With these arguments in mind, we present a deep learning method named Simpler Angle Predictor (SAP) to train simpler DNN models that enhance protein backbone angle prediction. We then empirically show that SAP significantly outperforms existing state-of-the-art methods on well-known benchmark datasets: for some types of angles, the differences are above 3 in mean absolute error (MAE). The SAP program along with its data is available from the website https://gitlab.com/mahnewton/sap.

## Introduction

Protein structure prediction (PSP) has been an unsolved problem for the last half century^1^. Three dimensional structures of most proteins depend on their amino acid (AA) sequences. The PSP problem is to determine the three dimensional structures of given proteins just from their amino acid sequences. The difficulties come from the inevitability of searching an astronomically large conformation space and from the absence of a highly accurate energy function to evaluate potential protein conformations^2^.

**Figure 1:**
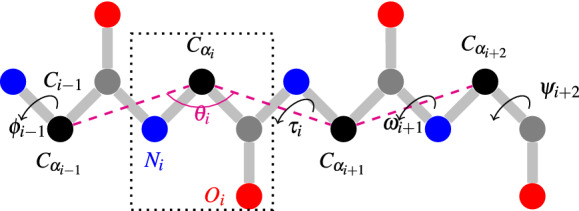
Backbone angles of a protein structure.

There are 20 types of amino acids. A protein might have any of the 20 types of amino acids any number of times in any order subject to stoichiometric constraints^3^. Each amino acid has three common atoms *N*, $C_\alpha$ and *C* among others. The *C* and *N* atoms of every two consecutive amino acids in a protein form a peptide bond and thus we obtain the backbone or main chain of the protein. As shown in Fig. 1, protein backbone structures can essentially be represented by dihedral angles $\phi$, $\psi$, and $\omega$, which are respectively defined by taking every four consecutive atoms from the sequence $C_{i-1}$, $N_i$, $C_{\alpha _i}$, $C_i$, $N_{i+1}$, $C_{\alpha _i}$. Typically $\omega$ is fixed at $180^\circ$ for majority proteins^4^, and so only $\phi$ and $\psi$ are to be determined. Besides being the parts of the main chain, each amino acid, starting from its $C_\alpha$ atom, has a side chain as well. The side chains have their own dihedral angles, but for this work we consider them to be out of scope. Once the backbone structures could be predicted with very high accuracy, side chain angles could be predicted or determined later. Besides $\phi$, $\psi$, and $\omega$ angles, as shown in Fig. 1, $\theta$ and $\tau$ angles provide an alternative representation for protein backbone structures. While $\theta$ is a planar angle defined by three consecutive $C_\alpha$ atoms, $\tau$ is a dihedral angle defined by four consecutive $C_\alpha$ atoms. Such a representation is actually possible because of the nearly constant distance between consecutive $C_\alpha$ atoms. While $\phi$ and $\psi$ are dihedral angles each involving four atoms from two consecutive residues, $\theta$ and $\tau$ involving three or four residues capture more local structures in a protein. In this work, we predict all the four types of backbone angles $\phi$, $\psi$, $\theta$ and $\tau$ for each protein in a given protein using deep neural networks (DNN).

Prediction of protein backbone structures is very important since both template-based and template-free protein structure prediction rely strongly on that^2,5^. From an abstraction based perspective, protein backbone structure prediction could be viewed as prediction of secondary structures (SSs). Protein secondary structure prediction has obtained significant success over the years through the use of various types of deep neural networks and their ensembles^6–12^ and ab initio methods^13^. For example, SSpro8^14^ achieves 79% accuracy on proteins with no homologs in the Protein Data Bank (PDB) and of 92% accuracy on proteins where homologs can be found in the PDB. However, this progress does not necessarily make backbone angle prediction trivial. With accurate SS predictions, one can obtain narrow ranges (about $20^\circ$) of $\phi$ and $\psi$ angles, but only for helices and sheets. For coils, $\phi$ and $\psi$ can take any value in $[-180, +180]$ and coils comprise about 40% residues in average proteins^15^. Moreover, errors in backbone angle prediction in one part of a protein has a cascaded effect in the construction of the entire protein structure. Overall, secondary structures, on one hand, are coarse-grained description of protein local structures in three (helices, sheets, and coils) or eight discrete states (including some variants of the three). On the other hand, secondary structures are somewhat arbitrarily defined with coils essentially having no well-defined structures. In contrast to secondary structures, backbone angles as being continuous variables can represent protein structures at greater accuracy levels. Moreover, predicted backbone dihedral angles, compared to the predicted secondary structures, have been found to be more useful in ab initio structure prediction or refinement by performing search^16,17^. Protein backbone angle prediction has improved over the years. A number of methods have been developed to predict $\phi$ and $\psi$ as both discrete^18,19^ and continuous^9,20–27^ labels.

Protein backbone angle prediction methods in recent years are mostly based on DNNs and their complex variants such as stacked sparse auto-encoder neural networks^23^, long short-term memory (LSTM) bidirectional recurrent neural networks (BRNNs)^6,25,27^, and Residual Networks (ResNets)^27^, and their ensembles^6,27^ or layered iterations^24^. In terms of input features, position specific scoring matrices (PSSM) produced by PSI-BLAST^28^ have been used by most methods^9,23–25,27^. Moreover, 7 physicochemical properties (7PCP) such as steric parameter (graph shape index), hydrophobicity, volume, polarisability, isoelectric point, helix probability, and sheet probability^29^ have been used as well^9,23–25,27^. Other input features that have been used include accessible surface area (ASA)^23^, Hidden Markov Model (HMM) profiles^9,27,30^ produced by HHBlits^31^, contact maps^27^, and PSP19^6^. In order to capture local structures around each given amino acids, sliding windows with various sizes have been used^23–25^. Moreover, to capture the non-local or long-range interactions among amino acids in a protein, the entire protein sequence has been used as features^9,24,26^ or convolutional neural networks (CNNs)^6,30^ or LSTM-BRNNs^25,27^ have been used. In terms of datasets to be used to evaluate the prediction models, we refer to four datasets: PISCES^32^, SPOT-1D^27,33^, PDB150^34^ and CAMEO93^35^. The first two datasets have respectively about 5.5K and 12.5K proteins with 1.2M and 2.7M residues. The last two datasets respectively have 150 and 93 proteins and have been used mainly for independent testing.

Given the literature explored above, we observe that in the protein backbone angle prediction research, there is an overall trend to employ more and more complex neural networks and then to throw more and more features to the neural networks. While more features might add more predictive power to the neural network, we argue that redundant features rather clutter the scenario and more complex neural networks then just counterbalance the noise. Similar results have been reported in other research areas. For example, in a Nature article in seismic aftershock prediction by deep learning methods^36^, a simple two-parameter logistic regression (that is, one neuron) is shown to have obtained the same performance as that of the 13,451-parameter DNN. From artificial intelligence and machine learning perspectives, problem representations and solution approaches do mutually interact and thus affect performance. Nevertheless, we also argue that comparatively simpler predictors can more easily be reconstructed than the more complex ones. With these arguments in mind, we present a deep learning method named Simpler Angle Predictor (SAP) to train simpler DNN models that enhance protein backbone angle prediction. We then empirically show that SAP significantly outperforms the existing state-of-the-art methods SPOT-1D and OPUS-TASS^6^ on well-known benchmark datasets: for ψ and τ, the differences are above 3 in mean absolute error (MAE). With an ensemble of several types of DNNs using many input features, SPOT-1D and OPUS-TASS are very complex prediction methods compared to the SAP, which uses just a fully connected DNN and a few input features. The SAP program along with its data is available from the website https://gitlab.com/mahnewton/sap.

## Methods

In this section, we describe the deep learning model proposed in this paper and the datasets used in this work.

### Input features

**Figure 2:**
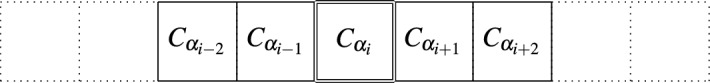
Sliding window of size 5: two residues on each side of a given residue.

As shown in Fig. 2, we use a sliding window of size *W*: up to $\tfrac{W}{2}$ amino acids at each side of a given amino acid. Depending on the window sizes, sliding windows can capture short or long range interactions between residues and secondary structures. Some backbone angle prediction methods that use recurrent neural networks (RNN) and CNNs take the whole protein sequences as input to capture interactions in the entire protein. However, with the absence of a firmly known energy function, it is not clear whether very long range interactions are really effective. So any choices regarding using sliding windows versus using entire proteins are to be made based on empirical evaluation. To make it clearer, in any distance-based energy components e.g. Lennard–Jones or charge-based potentials, the values are in effect zero after a certain distance. Moreover, if we look at the state-of-the-art backbone angle prediction method SPOT-1D, we see, besides using entire proteins, it still uses windowing to capture contact information. Our intent in this work is to explore simple models that can still achieve very good accuracy levels.

While window size effectively ensures context dependence of assumed local conformations, arguably there is not enough data in the training set, even in the protein data bank, to cover all possible combinations of amino acids (e.g. $20^5$) with a given window size (e.g. 5). So the context has to be captured via a 3-state a 8-state model that can specify the average range of angle values for each amino acid in a given protein. The data deficiency for larger windows even further spoils the training. In this work, for each amino acid, we consider one of the 8 values G, H, I, T, S, E, B, and C to represent predicted 8-state SS and then encode that using an one-hot vector. The 8-state SS prediction is obtained by running SSpro8^14^ on each protein. The training set of SSpro8 comprises 5772 proteins that are released before August 20, 2013. SSpro8 uses sequence similarity and sequence-based structural similarity in SS prediction and achieves respectively 92% and 79% accuracy on proteins with and without homologs in the PDB. On one hand, we have already discussed that these highly accurate SS predictions do not necessarily solve the backbone angle prediction problem when high quality protein structures are to be constructed. On the other hand, we note that we have removed all SSpro8’s training proteins from our training, validation, and tests sets and also use BLAST^28^ for this purpose with e-value 0.01. In this aspect, our method differs from the state-of-the-art backbone angle predictor SPOT-1D, which uses homologous sequences to generate its HMM-based features.

For each amino acid, we consider 20 values obtained from the PSSM matrix generated by three iterations of PSI-BLAST^28^ against the UniRef90 sequence database updated in April 2018. We also use 7PCP (seven physico-chemical properties) and ASA, and experiment with their various combinations. These features are very common in the literature.

In summary, we have $20 + 8 = 28$ PSSM and SS features plus various combinations of 7 or 1 feature values for 7PCP or ASA for each amino acid residue in each protein. This will be multiplied by the size of the sliding window used. We experiment with sliding windows of sizes 1, 5, 9, 13, 17, 21 as SPIDER^23^ tried up to size 21.

### Predicted outputs

We consider 4 outputs, one for each of $\phi$, $\psi$, $\theta$, and $\tau$ angles. Each $\phi$ and $\psi$ can be associated with exactly one residue or $C_\alpha$. A $\theta$ angle involving $C_{\alpha _{i-1}}, C_{\alpha _i}, C_{\alpha _{i+1}}$ is associated with $C_{\alpha _i}$. Similarly, a $\tau$ angle involving $C_{\alpha _{i-1}}, C_{\alpha _i}, C_{\alpha _{i+1}}, C_{\alpha _{i+2}}$ is associated with $C_{\alpha _i}$. In one set of experiments, we consider these angles directly, handling their periodicity ($-180^\circ$ to $180^\circ$) within the loss function of the DNN used. In another set of experiments, just like the state-of-the-art method SPOT-1D, we use both sine and cosine ratios for each of the 4 angles, and thus use 8 outputs. The trigonometric ratios handle the periodicity issue of the angles and the tangent values obtained from the sine and cosine values can give the predicted angle within $-180^\circ$ to $180^\circ$.

### DNN architecture

**Figure 3:**
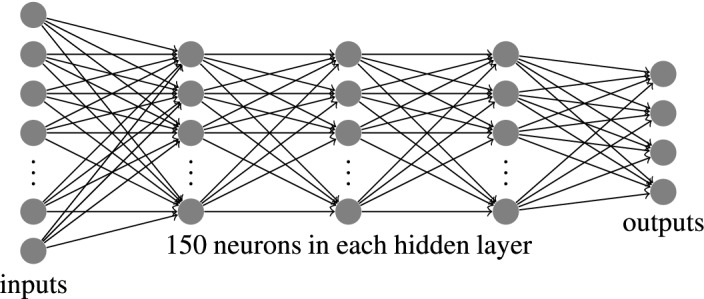
The fully connected deep neural network used in our method. It has three hidden layers, each having 150 neurons. The numbers of inputs and outputs could vary depending on the combinations of features used (e.g. PSSM plus SS and combinations of 7PCP and ASA) and the representation of the output angles (Direct Angles vs Trigonometric Ratios).

Figure 3 shows the DNN architecture used in our method. The DNN in fact is a fully connected neural network (FCNN) with three hidden layers, each having 150 neurons. This architecture is similar to that used in SPIDER^23^ and SPIDER2^24^. SPIDER2, however, uses a series of 3 DNNs feeding a previous DNN’s output as input to the next DNN. In our experiments, we have used only one DNN with three hidden layers, although we have trialled two and four hidden layers as well and showed the results later. The inputs and the outputs of the DNN are per amino acid basis. Depending on the size of the sliding window and the combinations of 7PCP and ASA, the input layer will have different numbers of inputs. The output layer has one output for each angle when we want to predict an angle directly. However, if we consider sine and cosine ratios of an angle and consequently later calculate the angle, then the output layer will have two outputs for each angle.

### DNN implementation

The DNN has been implemented in Python language using Keras library and SGD optimiser with momentum 0.9. The learning rate starts from 0.01 and if the loss function does not improve in 3 iterations, then learning rate is reduced by a factor 0.5 until it reaches $10^{-15}$. The activation function is linear in the output layer and sigmoid in the input and the hidden layers. The kernel initialiser is glorot_uniform. We run programs on NVIDIA Tesla V100-PCIE-32GB machines.

### Benchmark datasets

We briefly describe the dataset used by SPOT-1D^27^. This dataset has 12450 proteins that were culled from PISCES^32^ on Feb 2017 with the constraints of high resolution ($< 2.5A^\circ$), an R-free $< 1$, and a sequence identity cutoff of 25% according to BlastClust^28^. Among those proteins, 1250 proteins deposited after June 2015 were separated into an independent test set, leaving 11200 proteins, which were then randomly divided into a training set (10200 proteins) and a validation set (1000 proteins). Then, some proteins were removed to obtain efficient calculation. This reduced the training, validation, and independent test sets to 10029, 983, and 1213 proteins, respectively. In the SPOT-1D dataset, another independent test set was obtained from the PDB. These proteins were released between January 01, 2018 and July 16, 2018 and solved with resolution $< 2.5A^\circ$ and R-free $< 0.25$. In order to minimise evaluation bias associated with partially overlapping training data, proteins were removed for $>25\%$ sequence identity to structures released prior to 2018. This dataset was also filtered to remove redundancy at a 25% sequence identity cutoff and another 13 proteins with length $> 700$ were removed, leaving 250 high-quality, non-redundant targets. For convenience, these two independent test sets were denoted as TEST2016 (1213 proteins) and TEST2018 (250 proteins) as they were deposited between June 2015 and Feb 2017 and between Jan 2018 and July 2018, respectively.

We use the same dataset used by SPOT-1D^27^. However, we have performed additional filtering since it is not precisely clear to us how SPOT-1D handles the proteins that have mismatches in their amino acid sequences specified in various data source files (e.g. .t, .pssm, .dssp, and .fasta files). To be clearer, we have found that for some proteins, the amino acid sequence specified in one data source file has additional residues at the beginning or ending compared to that specified in another data source file. For such proteins, we have taken the part common in the amino acid sequences specified in various source files. However, when there is any mismatch at the middle of any two amino acid sequences specified in two different data source files for the same protein, we have removed the protein from the dataset. Also, we have removed proteins that have X in the secondary structure sequences in their corresponding DSSP files, although we do not use the secondary structure data from the DSSP files in our learning model. As mentioned before, apart from using subsets of features from SPOT-1D, we generate 8-state SS predictions using SSpro8^14^. The training set for SSpro8 comprised 5772 proteins released in the PDB before August 20, 2013. In order to avoid over-training with SSpro8 predictions as input of our method, we have removed 3259 proteins from SPOT-1D’s proteins using Blast^28^ against SSpro8’s training set with e-value 0.01. We show in Table 1 the numbers of proteins and residues in training, validation, and testing datasets, after performing the abovementioned filtering. As we can see later in Table 5, the remaining dataset after performing the filtering does not degrade the performance of SPOT-1D.

**Table 1:** Numbers of proteins and residues in training, validation, and testing datasets.

Datasets	Training	Validation	Testing	Total
Proteins	6721	667	1206	8594
Residues	1,670,605	165,530	282,461	2,118,596

27 testing proteins are in TEST2018 and 1179 are in TEST2016.

While our main training and test proteins are from the SPOT1D dataset, for further independent testing, we use PDB150^34^ and CAMEO93^35^ datasets. The PDB150 dataset contains 150 proteins released between February 1, 2019 and May 15, 2019. For each protein, PSI-BLAST^28^ was applied against the whole CullPDB^32^ dataset with e-value smaller than 0.005. The CAMEO93 dataset contains 93 proteins that are released between February 2020 and March 2020 and has been used by OPUS-TASS in its evaluation. For both datasets, we have applied 25% sequence similarity cutoff w.r.t. our and SSpro8’s training and validation datasets and also have removed proteins having X in their fasta file. For proteins with discontinuity in their amino acid sequences, we have considered largest segment of each protein so that our sliding window method can still be applied. At the end, we have obtained 71 and 55 proteins from the PDB150 and CAMEO93 datasets and we use them for independent testing of our method and the state-of-the-art method OPUS-TASS and compare their performance.

## Results

We compare various settings of SAP to find the best setting for each of the 4 types of angles to be predicted. This comparison helps us understand the impact of various features and encodings. Then, we compare the best settings with the current state-of-the-art predictors. Moreover, we show various other analyses of the results obtained for the best settings.

### Calculating absolute errors

For each predicted angle *P* against the actual angle *A*, we calculate the difference $D = |P - A|$. Then, we take AE = min(D,|360−D|) as the absolute error (AE) for that predicted angle. This addresses the periodicity issue that each angle must be in the range $-180^\circ$ to $180^\circ$. When angles are predicted directly, we implement the AE calculation within the loss function for the training and validation, and also later for testing. When we use sine and cosine ratios, then we calculate AE only during testing. In all cases, the angles that are not defined for the amino acids at the beginning or ending of the proteins are ignored.

### Determining best settings

We run 96 settings of SAP. All of these settings having 20 PSSM and 8 SS hot-vector features. The 96 settings are obtained by using or not using ASA, by using or not using 7PCP, by using range-based or Z-score based normalisation for input feature encoding, by using 6 window sizes (1, 5, 9, 13, 17, 21), and by using direct angles or trigonometric ratios to encode output angles. However, Table 2 presents performance of 16 settings only, selecting the best window size for each combination of the other parameters. From these results, it appears that window sizes 5 and 9 in most cases lead to better performances. Moreover, prediction of direct angles is better than that of trigonometric ratios. While not using ASA appears to be better than using, in contrast, using 7PCP appears to be better than not using. Overall, the best SAP setting is using 7PCP, range-based normalisation, direct angle prediction, and window size 5. Henceforth, we use this setting in further analysis.

**Table 2:** Performance of SAP settings on 1206 testing proteins. In the table, column ASA denotes whether accessible surface area is used (Yes/No), column 7PCP denotes whether 7 physicochemical properties are used (Yes/No), column OR denotes output representation is in direct angles (D) or trigonometric ratios (R), column NM denotes normalisation method for input feature encoding is [0,1] range based (R) or Z-score based (Z), WS denotes the best size of the sliding window. Note that the emboldened cells denote the best performance for each combination of ASA and 7PCP while the boxed plus emboldened cells in each respective column denote the best performance among all SAP settings.

Features		Encoding		$\phi$ MAE			$\psi$ MAE			$\theta$ MAE			$\tau$ MAE		
ASA	7PCP	OR	NM	WS	Test	Valid	WS	Test	Valid	WS	Test	Valid	WS	Test	Valid
N	N	D	R	5	17.14	17.66	5	20.19	20.48	5	6.40	6.49	5	22.74	23.02
			Z	5	**16.97**	**17.49**	5	**19.99**	**20.24**	5	**6.38**	**6.47**	5	22.44	22.68
		R	R	5	18.05	18.58	5	21.58	21.97	5	6.70	6.81	5	24.44	24.71
			Z	9	17.07	17.57	5	20.14	20.43	9	**6.38**	6.48	5	**22.39**	**22.59**
Y	N	D	R	9	**16.08**	**16.51**	9	**18.85**	**18.91**	9	**6.11**	**6.17**	9	21.83	21.35
			Z	5	16.44	16.55	5	19.36	19.56	5	6.23	6.18	9	21.52	21.63
		R	R	13	17.04	17.54	13	19.15	19.20	13	6.38	6.18	13	22.39	22.06
			Z	9	16.41	16.78	9	19.26	19.94	9	6.17	6.33	9	**21.15**	**21.28**
N	Y	D	R	5	$\overline{\underline{|\mathbf{15.65}|}}$	$\overline{\underline{|\mathbf{16.04}|}}$	5	$\overline{\underline{|\mathbf{18.59}|}}$	$\overline{\underline{|\mathbf{18.80}|}}$	5	$\overline{\underline{|\mathbf{6.07}|}}$	$\overline{\underline{|\mathbf{6.16}|}}$	5	$\overline{\underline{|\mathbf{21.03}|}}$	$\overline{\underline{|\mathbf{21.18}|}}$
			Z	5	16.42	16.84	5	19.59	19.84	5	6.32	6.41	5	22.23	22.47
		R	R	9	17.49	17.95	9	21.51	21.88	9	6.68	6.79	9	24.34	24.57
			Z	13	16.31	16.66	13	19.68	19.87	13	6.30	6.37	13	22.08	22.23
Y	Y	D	R	9	**15.79**	**16.13**	9	**18.84**	**18.85**	5	**6.12**	**6.17**	5	21.49	21.89
			Z	9	15.87	16.55	5	18.91	**18.85**	5	6.16	6.19	5	**21.12**	21.71
		R	R	9	16.15	16.85	9	19.30	19.91	9	6.23	6.20	9	21.63	21.71
			Z	9	16.70	16.51	9	18.86	18.91	9	6.17	6.20	9	21.74	**21.65**

It is worth noting here that in our observation, training a DNN simultaneously for several outputs is not much different from training the DNN separately for each output in terms of the accuracy level obtained for each output.

All results presented in Table 2 are for DNNs having 3 hidden layers. The choice of the number of layers was inspired by SPIDER^23^. However, in Table 3, we show the performance of the best SAP setting when run with DNNs having 2 and 4 hidden layers. In most cases DNNs having 3 hidden layers obtain the best results (shown in bold in Table 3); where this is not the case, DNNs with three hidden layers are a close second (shown in italics in Table 3), with the difference being < 0.09. So for the rest of the paper, we have chosen the DNN with 3 hidden layers as the selected SAP setting.

**Table 3:** Performance of the best SAP setting when the numbers of hidden layers in the DNNs are varied.

Hidden	$\phi$ MAE		$\psi$ MAE		$\theta$ MAE		$\tau$ MAE	
Layer	Test	Valid	Test	Valid	Test	Valid	Test	Valid
2	*15.70*	*16.09*	*18.65*	**18.71**	**6.05**	**6.14**	*21.04*	21.20
3	**15.65**	**16.04**	**18.59**	*18.80*	*6.07*	*6.16*	**21.03**	**21.18**
4	15.72	16.12	18.71	18.91	6.11	6.21	21.18	21.33

### Performing cross-validation

When we train a DNN, we specify the validation set. Consequently, the MAE values for the validation set as well as for the testing set for each SAP setting are shown in Table 2. In Table 4, we again show the MAE values but only for the best setting of SAP. However, to check the robustness of SAP, we perform 10-fold cross-validation, where the training and validation sets are merged. The merged proteins are then randomly divided into 10 folds. Then, 9 out of 10 folds are used in turn for training while the remaining one is used for testing. Table 4 shows the MAE value and the standard deviation of MAEs (SDMAE) for each type of angles to be predicted. As one can see, the small differences between MAE values and the small SDMAE values observed in the table shows the consistency and robustness of SAP.

**Table 4:** Average performance of the best setting of SAP after 10-fold cross validation is performed.

Dataset	Measure	$\phi$	$\psi$	$\theta$	$\tau$
Validation	MAE	16.04	18.80	6.16	21.18
Testing	MAE	15.65	18.59	6.07	21.03
10-Fold	MAE	16.14	18.82	6.33	21.31
10-Fold	SDMAE	0.24	0.09	0.08	0.21

### Comparison with state-of-the-art predictors

We mainly compare the performance of SAP with that of SPIDER2^24^, SPOT-1D^27^, and OPUS-TASS^6^ in Table 5. We have run these systems on the testing dataset that is used in this work and that is a subset of the SPOT-1D dataset because of more rigorous filtering. Moreover, we use 71 and 55 proteins from PDB150^34^ and CAMEO93^35^ datasets after performing filtering as mentioned before. However, we also compare SAP’s performance with that of SPIDER2, SPOT-1D, and OPUS-TASS as they are reported in the respective publications. Below we briefly describe SPIDER2, SPOT-1D, and OPUS-TASS.

1. **SPIDER2** is similar to SAP in that both use similar FCNN and similar features. SPIDER2 uses three DNNs of its precursor SPIDER^23^ in a series where the output of a previous DNN is fed as input to the next DNN in the series. Like SAP, SPIDER uses FCNN with 3 hidden layers each with 150 neurons. However, SPIDER uses stacked sparse auto-encoder for weight initialisation and 0-1 range normalisation for input values. SPIDER’s input features are PSSM, 3-state predicted SS, ASA, and 7PCP and the outputs are represented by trigonometric ratios. The window size is 21 in SPIDER and 17 in SPIDER2. SPIDER and SPIDER2 use the PISCES^32^ dataset, which has 5840 proteins.
2. **SPOT-1D** is a recent protein backbone angle predictor. It uses an ensemble of 9 long short term memory (LSTM) bidirectional recurrent neural networks (BRNNs) and Residual Networks (ResNets). SPOT-1D’s input features are PSSM, Hidden Markov Model (HMM), 7PCP, and contact maps. SPOT-1D obtains its predicted contact maps from SPOT-Contact^33^. SPOT-1D then uses windowing of the predicted contact maps. Further, SPOT-1D generates HMM profiles that include information about homologous sequences. For this, SPOT-1D uses HHBlits^31^ with the Uniprot sequence profile database from October 2017. SPOT-1D’s inputs are mapped in the range of [0, 1] and the outputs are represented by trigonometric ratios. SPOT-1D’s dataset is a superset of SAP’s dataset.
3. **OPUS-TASS** is the current state-of-the-art protein backbone angle predictor and predicts $\phi$ and $\psi$ only. Its architecture consists of CNN layers, LSTM layers, and Transformer^37^ layers. It uses an input feature named PSP19^38^, which classifies 20 residues into 19 rigid-body blocks depending on their local structures. It also introduces a new constrained/output feature named CSF3^39^, which is a local backbone structure descriptor. Further, it uses a multi-task learning strategy^40^ to maximise generalisation of the neural network and an ensemble of neural networks for further improvement.

Since SPOT-1D and OPUS-TASS show their performance on two subsets namely TEST2016 and TEST2018 of the testing proteins, we also do the same although we show the accumulated results for all testing proteins. Notice from the table that SAP significantly outperforms both SPOT-1D and OPUS-TASS in all cases. We have performed t-tests to compare the performances of SPOT-1D and OPUS-TASS with SAP and the *p* values are $< 0.01$ in all cases, indicating the differences are statistically significant. The differences are really huge for $\psi$ and $\tau$. These results demonstrate the effectiveness of SAP in enhancing protein backbone angle prediction accuracy.

Although our results are in Table 5, to test the generality of performance of SAP over other datasets, we have run SAP on 71 proteins of PDB150 dataset and 55 proteins of CAMEO93 datasets. In Table 6, we also compare SAP’s performance with SPOT-1D’s performance on the PDB150 proteins and with OPUS-TASS’s performance on the CAMEO93 proteins. The performance of various methods are rather mixed here. We have performed t-tests to compare the performances of SPOT-1D and OPUS-TASS with SAP and the p values are < 0.05 in all cases, indicating the differences are statistically significant.

**Table 5:** Performances of SPIDER2, SPOT-1D, SAP, and OPUS-TASS on our testing dataset and its subsets TEST2016 and TEST2018. The emboldened values are the winning numbers for the corresponding types of angles and datasets. OPUS-TASS does not predict $\theta$ and $\tau$ angles while the other three methods predict all four types of angles.

Results below are as we run all of the systems on our datasets							
Dataset	Proteins	Residues	Method	$\phi$ MAE	$\psi$ MAE	$\theta$ MAE	$\tau$ MAE
TEST2016	1179	278553	SPIDER2	18.93	30.14	8.15	32.13
			SPOT-1D	16.23	23.23	6.77	24.58
			OPUS-TASS	15.75	22.41	–	–
			SAP	**15.66**	**18.62**	**6.08**	**21.05**
TEST2018	27	3908	SPIDER2	18.51	28.78	7.80	30.35
			SPOT-1D	16.07	22.66	6.51	23.54
			OPUS-TASS	15.62	21.96	–	–
			SAP	**14.60**	**16.75**	**5.60**	**19.28**
Testing	1206	282461	SPIDER2	18.92	30.12	8.15	32.11
			SPOT-1D	16.23	23.22	6.77	24.57
			OPUS-TASS	15.74	22.41	–	–
			SAP	**15.65**	**18.59**	**6.07**	**21.03**

Results below are as they are reported in the respective publications							
Dataset	Proteins		Method	$\phi$ MAE	$\psi$ MAE	$\theta$ MAE	$\tau$ MAE
PISCES-test	1199		SPIDER2	19.7	30.3	8.2	32.6
TEST2016	1213		SPOT-1D	16.27	23.26	6.89	25.38
			OPUS-TASS	15.78	22.46	–	–
TEST2018	250		SPOT-1D	16.89	24.87	6.91	25.94
			OPUS-TASS	16.40	24.06	–	–

**Table 6:** Performances of SPIDER2, SPOT-1D, OPUS-TASS, and SAP on filtered PDB150 and CAMEO93 proteins. The emboldened values are the winning numbers for the corresponding types of angles and datasets. OPUS-TASS does not predict $\theta$ and $\tau$ angles while the other three methods predict all four types of angles.

Results below are as we run all of the systems on our datasets							
Dataset	Proteins	Residues	Method	$\phi$ MAE	$\psi$ MAE	$\theta$ MAE	$\tau$ MAE
PDB150	71	11547	SPIDER2	20.98	32.32	8.39	53.46
			SPOT-1D	**18.32**	**24.43**	**6.85**	52.58
			SAP	19.29	26.37	7.20	**51.89**
CAMEO93	55	13872	SPIDER2	20.05	31.80	8.34	33.83
			OPUS-TASS	**16.76**	**24.04**	–	–
			SAP	20.24	31.02	**7.87**	**32.69**

Results below are as they are reported in the respective publications							
Dataset	Proteins		Method	$\phi$ MAE	$\psi$ MAE	$\theta$ MAE	$\tau$ MAE
CAMEO	93		SPOT-1D	16.89	23.02		
			OPUS-TASS	16.56	22.56		

### Comparison on protein length groups

In Table 7, we compare the performance of SAP, OPUS-TASS, SPOT-1D, and SPIDER2 when our testing proteins are grouped based on their lengths i.e. the number of amino acids each protein has. This is to observe how SAP’s performance varies with the increase of the protein length. From the table, we see that for all four types of angles, SAP’s prediction accuracy gradually decreases, with minor exceptions, as the protein length increases. When protein lengths are 300 or below (with minor exception for θ), the MAE values are less than the overall MAE values and for protein lengths above 300, the MAE values are greater than the overall MAE values. From the $\Delta$MAE values (i.e. how far from SAP’s MAE) of OPUS-TASS, SPOT-1D and SPIDER2, we see that with the increase of protein lengths, the performance difference increases; which essentially means compared to OPUS-TASS’s or SPOT-1D’s or SPIDER2’s performance, SAP’s performance rather gets better.

**Table 7:** Performance of SAP, OPUS-TASS, SPOT-1D, and SPIDER2 when our testing proteins are grouped based on their lengths. In the table, $\Delta$MAE of a system (e.g. OPUS-TASS, SPOT-1D or SPIDER2) is its MAE minus the MAE of SAP. As such, the greater the value of $\Delta$MAE, the worse the performance of the system w.r.t. the performance of SAP.

Testing proteins		$\phi$				$\psi$				$\theta$			$\tau$		
		SAP	OPUS-TASS	SPOT-1D	SPIDER2	SAP	OPUS-TASS	SPOT-1D	SPIDER2	SAP	SPOT-1D	SPIDER2	SAP	SPOT-1D	SPIDER2
Length	Count	MAE	$\Delta$MAE	$\Delta$MAE	$\Delta$MAE	MAE	$\Delta$MAE	$\Delta$MAE	$\Delta$MAE	MAE	$\Delta$MAE	$\Delta$MAE	MAE	$\Delta$MAE	$\Delta$MAE
001–100	210	14.46	+ 0.11	+ 0.57	+ 3.03	17.88	+ 3.15	+ 3.71	+ 9.32	5.63	+ 0.53	+ 1.82	18.95	+ 2.68	+ 8.98
101–200	381	15.37	+ 0.02	+ 0.46	+ 3.08	18.40	+ 3.27	+ 3.93	+ 10.35	6.10	+ 0.55	+ 1.93	20.79	+2.63	+ 9.79
201–300	264	15.24	+ 0.25	+ 0.61	+ 3.17	18.02	+ 3.93	+ 4.66	+ 11.14	5.96	+ 0.71	+ 1.99	20.38	+ 3.50	+ 10.74
301–400	180	15.76	− 0.29	+ 0.30	+ 3.42	18.58	+ 3.06	+ 4.09	+ 11.70	6.12	+ 0.59	+ 2.10	21.43	+ 2.96	+ 11.23
401–500	102	16.06	+ 0.34	+ 0.87	+ 3.53	18.98	+ 4.76	+ 5.37	+ 12.91	6.09	+ 0.86	+ 2.32	21.49	+ 4.28	+ 12.36
501–800	69	16.52	+ 0.25	+ 0.81	+ 3.29	19.64	+ 4.75	+ 5.89	+ 12.77	6.29	+ 0.89	+ 2.20	22.04	+ 5.22	+ 12.44
Overall	1206	15.65	+ 0.09	+ 0.58	+ 3.27	18.59	+ 3.82	+ 4.63	+ 11.53	6.07	+ 0.70	+ 2.08	21.03	+ 3.54	+ 11.08

### Comparison on secondary structure groups

**Figure 4:**
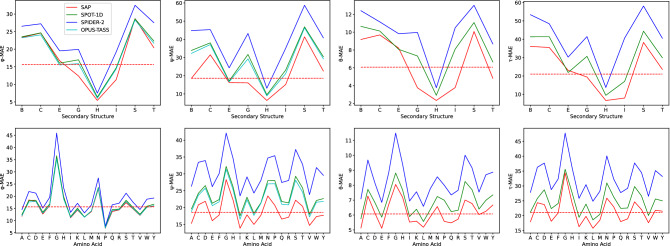
Performance of SAP, OPUS-TASS, SPOT-1D, SPIDER2 on the testing proteins when residues are grouped based (Top Four) on their SS types and (Bottom Four) on their AA types. In the charts, y-axis shows MAE values and x-axis shows SS or AA types. The dashed horizontal line in each chart shows the overall MAE value for SAP.

Table 8 (Left) shows the residue distribution over the testing proteins when the residues are grouped on their SS types. Types C, E, H, S and T are the most frequent groups. Figure 4 (Top Four) shows the MAE values of SAP, OPUS-TASS, SPOT-1D, and SPIDER2 when the residues are grouped on their SS types. From the charts, frequent SS type H appears to have the best MAE values while other frequent SS types C, E, and S have significantly worse MAE values than the overall MAE values.

**Table 8:** Residue distribution over the testing proteins when residues are grouped on their (Left) SS and (Right) AA types. Also, on the left, typical ranges suggested for the torsion angles $\phi$ and $\psi$ for various secondary structures^41^.

SS	Residues	Percentage	$\phi$ Range	$\psi$ Range
B	2955	1.05	[− 130, − 110]	[110, 130]
C	56,250	19.91	[− 180, + 180]	[− 180, + 180]
E	61,041	21.61	[− 130, − 110]	[110, 130]
G	10,581	3.75	[− 59, − 39]	[− 36, − 16]
H	96,993	34.34	[− 67, − 47]	[− 57, − 37]
I	47	0.02	[− 67, − 47]	[− 80, − 60]
S	22,984	8.14	[− 180, + 180]	[− 180, + 180]
T	31,610	11.19	[− 180, + 180]	[− 180, + 180]
Total	282,461	100.00		

AA	Residues	Percentages		
A	22,406	7.93		
C	3874	1.37		
D	16,697	5.91		
E	18,752	6.64		
F	12,022	4.26		
G	19,593	6.94		
H	6904	2.44		
I	16,152	5.72		
K	16,024	5.67		
L	26,909	9.53		
M	5963	2.11		
N	12,161	4.31		
P	12,752	4.51		
Q	10,567	3.74		
R	14,619	5.18		
S	17,387	6.16		
T	15,492	5.48		
V	19,622	6.95		
W	4170	1.48		
Y	10,395	3.68		
Total	282,461	100.00		

### Comparison on amino acid groups

Table 8 (Right) shows the residue distribution over the testing proteins when the residues are grouped on their AA types. Types A, D, E, G, I, K, L, P, R, S, T, and V are the most frequent groups having at least 4.5% residues. Figure 4 (Bottom Four) shows the MAE values of SAP, OPUS-TASS, SPOT-1D, and SPIDER2 when the residues are grouped on their AA types. From the charts, frequent AA types A, E, I, L appear to have the best MAE values in all 4 types of angles. Among other frequent AA types C, D, G have worse MAE values than the overall MAE values in some types of angles.

### Using angle ranges from predicted secondary structures

Given the SS predictions and their suggested ranges of $\phi$ and $\psi$ values as shown in Table 8 left, particularly for helices (G, H, I) and sheets (B, E), one might just use the mid values of the respectives ranges as the predicted values and expect an MAE of about 10 for the respective SS type. When we do that for the residues that belong to SS types G, H and I, we get MAE values respectively 27.71, 9.12, and 22.04 for $\phi$ and 18.71, 8.83, 21.17 for $\psi$. In contrast, the MAE values for SAP predictions are respectively 12.40, 5.43, 11.34 for $\phi$ for SS types G, H, and I, and 16.08, 6.40, 15.16 for $\psi$. The situations worsens for sheets such as SS types B and E. These results clearly show that just achieving higher accuracy in SS prediction would not be sufficient for backbone angle prediction.

### Comparison of angle distributions

**Figure 5:**
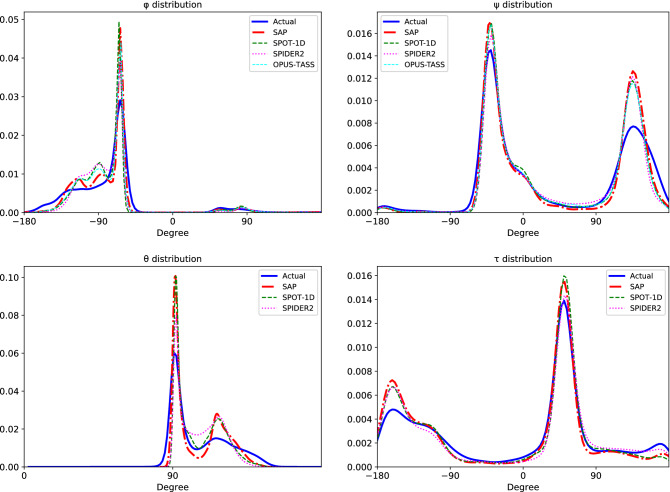
Distributions of actual angles of testing proteins and predictions of SAP, OPUS-TASS, SPOT-1D, and SPIDER2.

Figure 5 shows the distributions of the actual angles and predicted values obtained from SAP, OPUS-TASS, SPOT-1D, and SPIDER2. As we can see from the charts, the distribution of values predicted by SAP aligns very well with the distribution of the actual values. The peaks and troughs of the distributions align quite well, even multiple peaks and troughs are captured well. While the peaks of the predicted distributions are larger and narrower than those of the actual distributions, the troughs of the predicted distributions are rather smaller and wider than those of the actual distributions. When SAP’s curves are compared with OPUS-TASS’s, SPOT-1D’s and SPIDER2’s, we see SAP’s curves are occasionally closer to the curves for the actual values. We also see that the distributions of $\phi$ and $\psi$ angles for are OPUS-TASS and SPOT-1D are almost similar. Notice that the largest peaks of the predicted values are higher than the largest peaks of the actual values. One noticeable fact is in the $\theta$ chart: there are actual values between 0 and 90 although with almost zero probability, and these values are not much captured by the predictors. Overall, there is a tendency to predict the peak values with probabilities larger than that of the actual values.

### Protein structure generation and refinement

**Figure 6:**
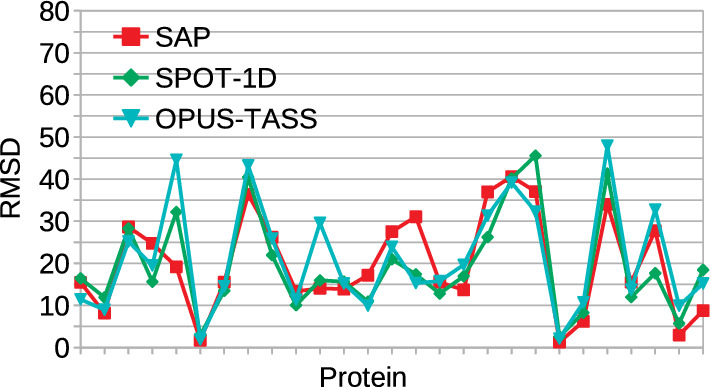
RMSD values for SAP, SPOT-1D, and OPUS-TASS on TEST2018 proteins.

Given the improvement in angle prediction accuracy, an interesting question is as follows: “Can predicted angles be directly employed in building accurate protein structures?” The direct answer to this question is yes if we reach to a very high accuracy level. This is actually the aim of this study to enhance the performance gradually to the level that would predict protein structures with very high accuracy; which is very challenging. Given the 27 proteins in our TEST2018 set, we have tried to generate entire protein structures from the predicted values obtained from SAP, OPUS-TASS, and SPOT-1D, and assuming $\omega =180^\circ$ and standard bond distances. From Fig. 6, we can see very high root mean square distance (RMSD) for more proteins and only for 2–3 proteins, RMSD values are less than 6$A^\circ$, a distance considered to be practically meaningful. Although this is the case with protein structure generation, for structure refinement via ab initio structure sampling and evaluation by using perturbation techniques would obtain significant help. This is because given a prediction $\rho$ and estimated error $\epsilon$, with some level of certainty, one can focus searching within the region $[\rho -\epsilon , \rho +\epsilon ]$. These soft constraints can thus reduce the search space significantly. With more proteins having more dihedral angles predicted with less absolute errors, *ab initio* or refinement search for protein structures would be benefited more from SAP’s prediction than OPUS-TASS’s or SPOT-1D’s.

### Comparison on correct prediction per protein

**Figure 7:**
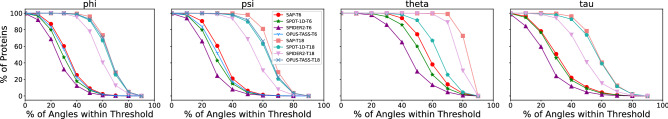
Percentages of proteins (y-axis) that have a given percentage of residues (x-axis) with AE at most a given threshold *T* where *T* is 6 and 18 and are denoted by T6 and T18. The lower the threshold, the better the prediction quality.

Having the discussion regarding structure generation and refinement, we compare SAP, OPUS-TASS, and SPOT-1D on what portions of the angles of the proteins are predicted within certain error levels. Figure 7 shows the percentages of proteins that have a given percentage of particular angles with absolute errors at most a given threshold. We choose the threshold values to be 6 and 18 in the charts. Notice that SPOT-1D’s and OPUS-TASS’s performances are very close in the charts for $\phi$ and $\psi$. Moreover, SAP outperforms the other three methods in all angles in all threshold levels.

## Conclusions

Input features and neural network architectures interact with each other when employed in prediction systems. Consequently, inclusions of just more features might cause cluttering and the complex networks might then be needed to counterbalance. In the protein backbone angle prediction research, the existing state-of-the-art prediction method uses ensembles of several types of deep neural networks and a number of features. In this paper, we present simpler deep neural network models for protein backbone angle prediction. Our models use fewer features and simpler neural networks but on a standard benchmark dataset obtain significantly better mean absolute errors than the state-of-the-art predictor. Our program named Simpler Angle Predictor (SAP) along with its data is available from the website https://gitlab.com/mahnewton/sap.
