# Author Correction: Enhancing protein backbone angle prediction by using simpler models of deep neural networks

**DOI:** 10.1038/s41598-021-96666-0

**Published:** 2021-09-06

**Authors:** Fereshteh Mataeimoghadam, M. A. Hakim Newton, Abdollah Dehzangi, Abdul Karim, B. Jayaram, Shoba Ranganathan, Abdul Sattar

**Affiliations:** 1 School of Information and Communication Technology, Griffith University, Nathan, QLD Australia; 2 Institute of Integrated and Intelligent Systems, Griffith University, Nathan, QLD Australia; 3 Department of Computer Science, Rutgers University, Camden, NJ USA; 4 Center for Computational and Integrative Biology, Rutgers University, Camden, NJ USA; 5 Department of Chemistry and School of Biological Sciences, IIT Delhi, Delhi, India; 6 Department of Chemistry and Biomolecular Sciences, Macquarie University, Macquarie Park, NSW Australia

Correction to: *Scientific Reports* 10.1038/s41598-020-76317-6, published online 10 November 2020

The original version of this Article contained errors.

Following the publication of this Article the Authors detected programming errors that affected the accuracy of some of the results:

- Instead of atan2() function to compute arctangent, atan() function was used in some cases. atan() function returns angles only in − 90 to 90 degree range while atan2() function returns in − 180 to 180 degree range taking the quadrant information into account. Backbone angles predicted are in the range of − 180 to 180. All calculations were now re-done using atan2() function.
- Difference between two angles +190 and − 175 is 365. However, considering the periodicity of − 180 to 180, a difference can only be within 0 to 180. As such the difference of 365 was given as 5. In another case the difference between 170 and − 185 is 355 which is also given as 5. To compute the difference correctly in both cases, an abs() function should be used in the formula min(D, abs(360-D)). This was previously omitted, but calculations were re-done now using the correct version of the formula.
- In MAE computation function, a parameter was passed by reference. However, the parameter was mistakenly assumed to be passed by value and was changed within the function with the understanding that the change does not affect outside the function. This assumption was incorrect, as the change within the function affects outside the function. Calculations were now re-done to reflect this.

As a result of the correction for these programming errors, in the Abstract,

“We then empirically show that SAP can significantly outperform existing state-of-the-art methods on well-known benchmark datasets: for some types of angles, the differences are 6–8 in terms of mean absolute error (MAE).”

now reads:

“We then empirically show that SAP significantly outperform existing state-of-the-art methods on well-known benchmark datasets: for some types of angles, the differences are above 3 in mean absolute error (MAE).”

In the Introduction,

“We then empirically show that SAP can significantly outperform the existing state-of-the-art methods SPOT-1D and OPUS-TASS^6^ on well-known benchmark datasets: for *ψ* and *τ* , the differences are 6–8 in terms of mean absolute error (MAE).”

now reads:

“We then empirically show that SAP significantly outperforms the existing state-of-the-art methods SPOT-1D and OPUS-TASS^6^ on well-known benchmark datasets: for *ψ* and *τ* , the differences are above 3 in mean absolute error (MAE).”

In the Results section, under the subheading ‘Calculating Absolute Errors’,

“Then, we take *AE* = min(*D*,360−*D*) as the absolute error (AE) for that predicted angle.”

now reads:

“Then, we take AE = min(D,|360−D|) as the absolute error (AE) for that predicted angle.”

In the Results section, under the subheading ‘Determining Best Settings’,

“Moreover, prediction of trigonometric ratios is better for *ψ* while prediction of direct angles is better for $\phi$ , *θ* , and $\tau$ . While not using ASA appears to be better than using, in contrast, using 7PCP appears to be better than not using. Overall, the best SAP settings to predict the 4 types of angles are listed below. Henceforth, we use these angle specific settings in further analysis.”

now reads:

“Moreover, prediction of direct angles is better than that of trigonometric ratios. While not using ASA appears to be better than using, in contrast, using 7PCP appears to be better than not using. Overall, the best SAP settings is using 7PCP, range-based normalisation, direct angle prediction, and window size 5. Henceforth, we use this setting in further analysis.”

In the same section, the following text was removed:

- $\phi$: 7PCP, range-based normalisation, direct angle prediction, and window size 5
- *ψ*: 7PCP, z-score based normalisation, trigonometric ratio prediction, and window size 13
- *θ*: 7PCP, range-based normalisation, direct angle prediction, and window size
- *τ*: 7PCP, range-based normalisation, direct angle prediction, and window size 5

In the Results section, still under the subheading ‘Determining Best Settings’,

“However, in Table 3, we show the performance of the best angle specific SAP settings when run with DNNs having 2 and 4 hidden layers. In most cases DNNs having 3 hidden layers show the best results (shown in bold in Table 3); where this is not the case, DNNs with three hidden layers are a close second (shown in italics in Table 3), with the difference being < 0.05. So for the rest of the paper, we have chosen DNNs with 3 hidden layers as the selected SAP settings”

now reads:

“However, in Table 3, we show the performance of the best angle specific SAP setting when run with DNNs having 2 and 4 hidden layers. In most cases DNNs having 3 hidden layers show the best results (shown in bold in Table 3); where this is not the case, DNNs with three hidden layers are a close second (shown in italics in Table 3), with the difference being < 0.09. So for the rest of the paper, we have chosen the DNN with 3 hidden layers as the selected SAP setting.”

In the Results section, under the subheading ‘Performing cross-validation’,

“In Table 4, we again show the MAE values but only for the best settings of SAP”

now reads:

“In Table 4, we again show the MAE values but only for the best setting of SAP”

In the Results section, under the subheading ‘Comparison with state-of-the-art predictors’

“Since SPOT-1D and OPUS-TASS show their performance on two subsets namely TEST2016 and TEST2018 of the testing proteins, we also do the same although we show the accumulated results for all testing proteins. Note that both SPOT-1D’s and OPUS-TASS’s performances are not worse than their reported values as shown in the bottom part of Table 5. Moreover, notice from the table that SAP significantly outperforms both SPOT-1D and OPUS-TASS in all cases.”

now reads:

“Since SPOT-1D and OPUS-TASS show their performance on two subsets namely TEST2016 and TEST2018 of the testing proteins, we also do the same although we show the accumulated results for all testing proteins. Notice from the table that SAP significantly outperforms both SPOT-1D and OPUS-TASS in all cases.”

In the Results section, still under the subheading ‘Comparison with state-of-the-art predictors’

“To test the generality of performance of SAP over other datasets, we have run SAP on 71 proteins of PDB150 dataset and 55 proteins of CAMEO93 datasets. In Table 6, we also compare SAP’s performance with SPOT-1D’s performance on the PDB150 proteins and with OPUS-TASS’s performance on the CAMEO93 proteins. Notice that SAP significantly outperforms SPOT-1D and OPUS-TASS in *ψ*, *θ*, and *τ* angles, but performs worse in $\phi$ prediction. We have performed t-tests to compare the performances of SPOT-1D and OPUS-TASS with SAP and the *p* values are < 0.01 in all cases, indicating the differences are statistically significant. Nevertheless, the margins in *ψ* and *τ* remain huge for SAP compared to the other methods.”

now reads:

“Although our results are in Table 5, to test the generality of performance of SAP over other datasets, we have run SAP on 71 proteins of PDB150 dataset and 55 proteins of CAMEO93 datasets. In Table 6, we also compare SAP’s performance with SPOT-1D’s performance on the PDB150 proteins and with OPUS-TASS’s performance on the CAMEO93 proteins. The performance of various methods are rather mixed here. We have performed t-tests to compare the performances of SPOT-1D and OPUS-TASS with SAP and the p values are < 0.05 in all cases, indicating the differences are statistically significant.”

In the Results section, under the subheading ‘Comparison on Protein Length Groups’,

“From the table, we see that for all four types of angles, SAP’s prediction accuracy gradually decreases as the protein length increases. When protein lengths are 300 or below, the MAE values are less than the overall MAE values and for protein lengths above 300, the MAE values are greater than the overall MAE values.”

now reads:

“From the table, we see that for all four types of angles, SAP’s prediction accuracy gradually decreases, with minor exceptions, as the protein length increases. When protein lengths are 300 or below (with minor exception for *ϑ*), the MAE values are less than the overall MAE values and for protein lengths above 300, the MAE values are greater than the overall MAE values.”

In the Results section, under the subheading ‘Using Angle Ranges from Predicted Secondary Structures’,

“When we do that for the residues that belong to SS types G, H and I, we get MAE values respectively 16.91, 8.78, and 24.02 for $\phi$ and 27.71, 9.12, 22.04 for *ψ*. In contrast the MAE values for SAP predictions are respectively 12.39, 5.43, 11.34 for $\phi$ for SS types G, H, and I, and 12.41, 5.73, 12.06 for *ψ*.”

now reads:

“When we do that for the residues that belong to SS types G, H and I, we get MAE values respectively 27.71, 9.12, and 22.04 for $\phi$ and 18.71, 8.83, 21.17 for *ψ*. In contrast the MAE values for SAP predictions are respectively 12.40, 5.43, 11.34 for $\phi$ for SS types G, H, and I, and 16.08, 6.40, 15.16 for *ψ*.”

In the Results section, under the subheading ‘Comparison of angle distributions’,

“Notice that the largest peaks of the predicted values are higher than the largest peaks of the actual values except in predicted *ψ* distributions of OPUS-TASS, SPOT-1D and SPIDER2. One noticeable fact in the *ψ* chart is OPUS-TASS, SPOT-1D and SPIDER2 predicted values are outside of [−90, 90] but actual values are roughly within the range. Another noticeable fact is in the *θ* chart: there are actual values between 0 and 90 although with almost zero probability, and these values are not much captured by the predictors.”

now reads:

“Notice that the largest peaks of the predicted values are higher than the largest peaks of the actual values. One noticeable fact is in the $\theta$ chart: there are actual values between 0 and 90 although with almost zero probability, and these values are not much captured by the predictors.”

Finally, in the Results section, under the subheading ‘Comparison on Correct Prediction Per Protein’,

“We choose the threshold values to be 6 and 18 in the charts because SAP’s minimum and maximum MAE values are close to 6 and 18 respectively, for example for *θ* and *τ*.”

now reads:

“We choose the threshold values to be 6 and 18 in the charts.”

Additionally, Figure 4, Figure 5, Figure 6, and Figure 7 were corrected with the updated results for SAP. The original versions of these figures are reproduced below for the record.

**Figure 4:**
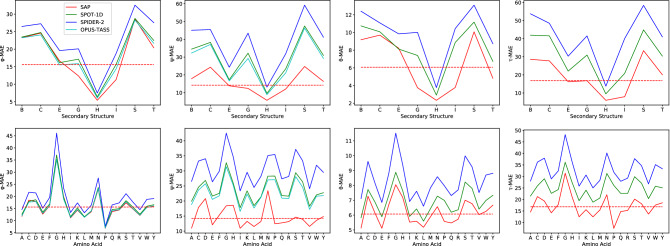
Performance of SAP, OPUS-TASS, SPOT-1D, SPIDER2 on the testing proteins when residues are grouped based (Top Four) on their SS types and (Bottom Four) on their AA types. In the charts, y-axis shows MAE values and x-axis shows SS or AA types. The dashed horizontal line in each chart shows the overall MAE value for SAP.

**Figure 5:**
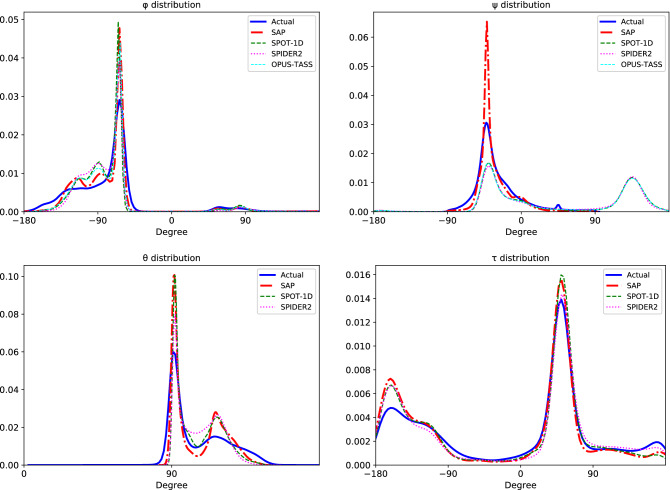
Distributions of actual angles of testing proteins and predictions of SAP, OPUS-TASS, SPOT-1D, and SPIDER2.

**Figure 6:**
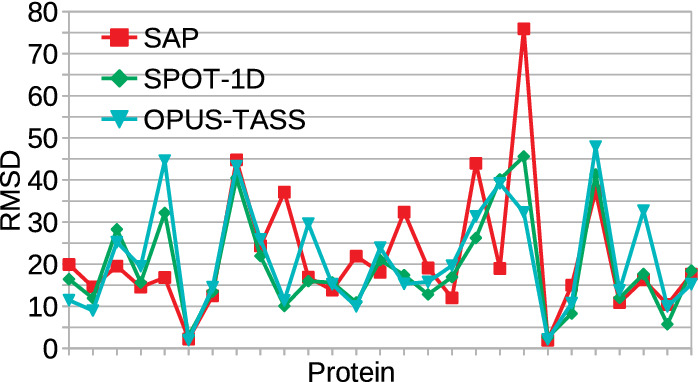
RMSD values for SAP, SPOT-1D, and OPUS-TASS on TEST2018 proteins.

**Figure 7:**
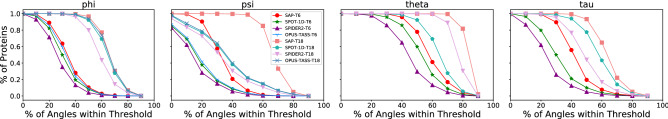
Percentages of proteins (y-axis) that have a given percentage of residues (x-axis) with AE at most a given threshold *T* where *T* is 6 and 18 and are denoted by T6 and T18. The lower the threshold, the better the prediction quality.

Furthermore, Table 2, Table 3, Table 4, Table 5, Table 6, Table 7 were corrected to reflect updated results for SAP. The original versions of these tables are reproduced below for the record.

**Table 2:** Performance of SAP settings on 1206 testing proteins. In the table, column ASA denotes whether accessible surface area is used (Yes/No), column 7PCP denotes whether 7 physicochemical properties are used (Yes/No), column OR denotes output representation is in direct angles (D) or trigonometric ratios (R), column NM denotes normalisation method for input feature encoding is [0,1] range based (R) or Z-score based (Z), WS denotes the best size of the sliding window. Note that the emboldened cells denote the best performance for each combination of ASA and 7PCP while the boxed plus emboldened cells in each respective column denote the best performance among all SAP settings.

Features		Encoding		*ϕ* MAE			*ψ* MAE			*θ* MAE			*τ* MAE		
ASA	7PCP	OR	NM	WS	Test	Valid	WS	Test	Valid	WS	Test	Valid	WS	Test	Valid
N	N	D	R	5	17.19	17.53	5	20.27	20.42	5	6.42	6.45	5	18.89	19.00
			Z	9	**16.79**	**17.13**	5	19.98	20.15	5	**6.37**	**6.40**	5	**17.94**	**17.91**
		R	R	17	37.39	37.75	17	15.90	16.10	17	31.30	31.37	17	25.36	25.59
			Z	17	34.76	35.14	17	**15.13**	**15.30**	9	30.54	30.69	9	23.70	23.87
Y	N	D	R	9	**18.17**	**18.51**	5	21.91	22.11	9	**6.72**	**6.77**	5	**21.25**	**21.35**
			Z	1	19.21	19.55	1	24.32	24.56	1	7.14	7.18	13	28.30	28.63
		R	R	9	36.03	36.54	5	15.80	16.00	5	31.14	31.18	9	24.89	25.06
			Z	9	33.47	33.78	9	**14.82**	**14.94**	9	30.24	30.33	9	22.65	22.81
N	Y	D	R	5			5	19.60	19.68	5			5		
			Z	1	18.77	19.00	1	24.77	24.98	1	7.24	7.27	17	29.94	30.13
		R	R	13	34.20	34.56	13	14.94	15.03	13	30.36	30.45	13	23.15	23.30
			Z	13	33.11	33.39	13			5	30.22	30.30	9	22.77	22.92
Y	Y	D	R	9	**18.79**	**18.13**	9	22.00	22.15	5	**7.21**	**7.27**	9	**21.00**	**21.09**
			Z	1	21.55	21.55	9	28.55	28.85	9	8.13	8.19	1	36.64	37.01
		R	R	5	35.46	35.85	5	**15.62**	**15.81**	5	31.06	31.20	5	24.46	24.71
			Z	9	33.08	33.51	5	15.67	15.81	5	30.10	30.20	9	22.58	22.65

**Table 3:** Performance of the best angle specific SAP settings when the numbers of hidden layers in the DNNs are varied.

Hidden	*ϕ* MAE		*ψ* MAE		*θ* MAE		*τ* MAE	
Layer	Test	Valid	Test	Valid	Test	Valid	Test	Valid
2	**15.62**	15.97	14.66	14.86	6.08	6.22	16.97	17.07
3	**15.62**	**15.57**	**14.23**	*14.55*	**6.07**	**6.19**	*16.85*	**16.94**
4	15.66	16.06	14.36	**14.52**	6.11	6.21	**16.84**	16.96

**Table 4:** Average performance of the best settings of SAP after 10-fold cross validation is performed.

Dataset	Measure	*ϕ*	*ψ*	*θ*	*τ*
Validation	MAE	15.57	14.55	6.19	16.94
Testing	MAE	15.62	14.23	6.07	16.85
10-Fold	MAE	16.54	14.94	6.33	17.71
10-Fold	SDMAE	0.25	0.07	0.08	0.22

**Table 5:** Performances of SPIDER2, SPOT-1D, SAP, and OPUS-TASS on our testing dataset and its subsets TEST2016 and TEST2018. The emboldened values are the wining numbers for the corresponding types of angles and datasets. OPUS-TASS does not predict *θ* and* τ* angles while the other three methods predict all four types of angles.

Results below are as we run all of the systems on our datasets							
Dataset	Proteins	Residues	Method	*ϕ* MAE	*ψ* MAE	*θ* MAE	*τ* MAE
TEST2016	1179	278553	SPIDER2	18.80	29.93	8.08	31.72
			SPOT-1D	16.12	23.07	6.71	24.27
			OPUS-TASS	15.75	22.41	–	–
			SAP	**15.63**	**14.25**	**6.07**	**16.87**
TEST2018	27	3908	SPIDER2	17.17	27.69	7.22	28.86
			SPOT-1D	15.05	22.17	6.12	22.78
			OPUS-TASS	15.62	21.96	–	–
			SAP	**14.58**	**13.22**	**5.60**	**15.22**
Testing	1206	282461	SPIDER2	18.52	29.55	7.94	31.24
			SPOT-1D	15.94	22.92	6.61	24.02
			OPUS-TASS	15.74	22.41	–	–
			SAP	**15.62**	**14.23**	**6.07**	**16.85**

Results below are as they are reported in the respective publications							
Dataset		Proteins	Method	*ϕ* MAE	*ψ* MAE	*θ* MAE	*τ* MAE
PISCES-test		1199	SPIDER2	19.7	30.3	8.2	32.6
TEST2016		1213	SPOT-1D	16.27	23.26	6.89	25.38
			OPUS-TASS	15.78	22.46	–	–
TEST2018		250	SPOT-1D	16.89	24.87	6.91	25.94
			OPUS-TASS	16.40	24.06	–	–

**Table 6:** Performances of SPIDER2, SPOT-1D, OPUS-TASS, and SAP on filtered PDB150 and CAMEO93 proteins. The emboldened values are the wining numbers for the corresponding types of angles and datasets. OPUS-TASS does not predict *θ* and *τ* angles while the other three methods predict all four types of angles.

Results below are as we run all of the systems on our datasets							
Dataset	Proteins	Residues	Method	*ϕ* MAE	*ψ* MAE	*θ* MAE	*τ* MAE
PDB150	71	14964	SPIDER2	20.63	32.54	8.48	33.56
			SPOT-1D	**18.37**	25.48	7.74	26.54
			SAP	19.69	**17.14**	**7.57**	**25.70**
CAMEO93	55	13872	SPIDER2	20.35	31.80	8.34	33.83
			OPUS-TASS	**16.76**	24.04	–	–
			SAP	20.23	**17.60**	**7.87**	**28.58**

Results below are as they are reported in the respective publications							
Dataset	Proteins	Method		*ϕ* MAE	*ψ* MAE	*θ* MAE	τ MAE
CAMEO	93	SPOT-1D		16.89	23.02		
		OPUS-TASS		16.56	22.56

**Table 7:** Performance of SAP, OPUS-TASS, SPOT-1D, and SPIDER2 when our testing proteins are grouped based on their lengths. In the table, ΔMAE of a system (e.g. OPUS-TASS, SPOT-1D or SPIDER2) is its MAE minus the MAE of SAP. As such, the greater the value of ΔMAE, the worse the performance of the system w.r.t. the performance of SAP. The horizontal lines in SAP columns split those columns such that the upper parts have MAE values less than the overall MAE values and the lower parts have MAE values greater (a slight exception for θ).

Testing proteins		*Φ*				*ψ*				*θ*			*τ*		
		SAP	OPUS- TASS	SPOT-1D	SPIDER2	SAP	OPUS-TASS	SPOT-1D	SPIDER2	SAP	SPOT-1D	SPIDER2	SAP	SPOT-1D	SPIDER2
Length	Count	MAE	∆MAE	∆MAE	∆MAE	MAE	∆MAE	∆MAE	∆MAE	MAE	∆MAE	∆MAE	MAE	∆MAE	∆MAE
001–100	210	14.43	+ 0.14	+ 0.34	+ 2.75	13.19	+ 7.84	+ 8.02	+ 13.53	5.63	+ 0.36	+ 1.61	15.19	+ 5.52	+ 11.55
101–200	381	15.33	+ 0.06	+ 0.35	+ 2.93	13.73	+ 7.94	+ 8.43	+ 14.77	6.09	+ 0.47	+ 1.82	16.54	+ 6.44	+ 13.42
201–300	264	15.21	+ 0.28	+ 0.56	+ 3.03	13.94	+ 8.01	+ 8.59	+ 14.99	5.96	+ 0.67	+ 1.93	16.25	+ 7.35	+ 14.46
301–400	180	15.73	− 0.26	+ 0.26	+ 3.36	14.29	+ 7.35	+ 8.30	+ 15.85	6.11	+ 0.57	+ 2.07	17.12	+ 7.08	+ 15.27
401–500	102	16.03	+ 0.37	+ 0.79	+ 3.44	14.70	+ 9.04	+ 9.45	+ 16.97	6.09	+ 0.81	+ 2.27	17.27	+ 8.33	+ 16.32
501–800	69	16.49	+ 0.28	+ 0.77	+ 3.25	15.22	+ 9.17	+ 10.21	+ 17.07	6.29	+ 0.86	+ 2.17	17.97	+ 9.15	+ 16.33
Overall	1206	15.62	+ 0.12	+ 0.41	+ 2.90	14.23	+ 8.18	+ 8.69	+ 15.32	6.07	+ 0.54	+ 1.87	16.85	+ 7.17	+ 14.39

Finally, legends of Table 3, 4, and 7 were also updated. In the legend of Table 3,

“Performance of the best angle specific SAP settings when the numbers of hidden layers in the DNNs are varied”

now reads:

“Performance of the best SAP setting when the numbers of hidden layers in the DNNs are varied”

In the legend of Table 4,

“Average performance of the best settings of SAP after 10-fold cross validation is performed.”

now reads:

“Average performance of the best setting of SAP after 10-fold cross validation is performed.”

and in the legend of Table 7,

“As such, the greater the value of ΔMAE, the worse the performance of the system w.r.t. the performance of SAP. The horizontal lines in SAP columns split those columns such that the upper parts have MAE values less than the overall MAE values (a slight exception for q) and the lower parts have MAE values greater (a slight exception for *θ*).”

now reads:

“As such, the greater the value of ΔMAE, the worse the performance of the system w.r.t. the performance of SAP.”

The original version of the Article has been corrected.

